# Supplementary material for: Ontology-guided clustering enables proteomic analysis of rare pediatric disorders
Source: EMBO Mol Med. 2025 May 27;17(7):1842–67. doi: 10.1038/s44321-025-00253-z (PMC12254340; doi:10.1038/s44321-025-00253-z)
Supplement: Supplementary file 8 — Expanded View Figures [file 44321_2025_253_MOESM8_ESM.pdf]

Expanded View Figures

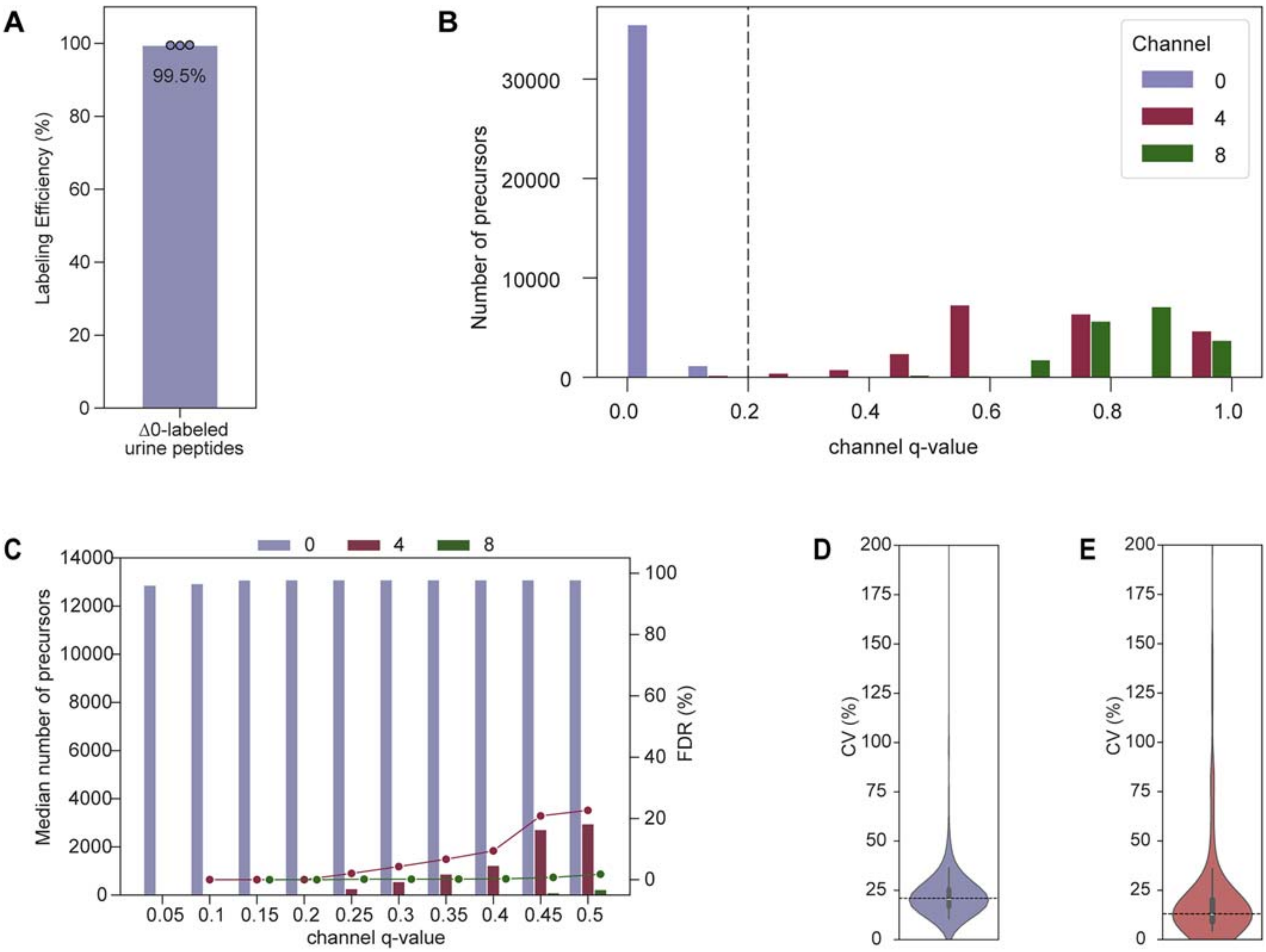

Figure EV1. Assessment of data quality in the urine and neat plasma proteomics datasets.

(**A**) Dimethyl labeling efficiency in the urine dataset, assessed by comparing the intensity ratios of Δ0-labeled peptides to all detected peptides in DDA mode across three technical replicates ($n = 3$). (**B**) Distribution of precursor identifications for a Δ0-labeled pooled urine sample analyzed through the mDIA workflow across three technical replicates ($n = 3$). (**C**) Median number of precursor identifications for each of the three dimethyl labeling channels (Δ0/Δ4/Δ8), calculated across three technical replicates for a Δ0-labeled pooled urine sample ($n = 3$). The secondary axis displays the percentage of false discovery rate (FDR), derived from the ratio of false precursor identifications in the Δ4 and Δ8 channels relative to the Δ0 channel. (**D**) Violin plot of analytical coefficients of variation (%CV) for protein groups identified in the reference channel of the urine proteomics dataset, filtered by a channel q-value < 0.2, across 553 technical replicates ($n = 553$). A horizontal dashed line indicates the median analytical CV of 21%. The internal boxplot shows the interquartile range (IQR), with whiskers extending to the most extreme point within 1.5 times the IQR. Outliers are not shown for clarity. (**E**) Violin plot of analytical coefficients of variation (%CV) for protein groups identified in the QC samples of the neat plasma proteomics dataset, filtered by a channel q-value < 0.2, across 112 technical replicates ($n = 112$). A horizontal dashed line indicates the median analytical CV of 13%. The internal boxplot shows the interquartile range (IQR), with whiskers extending to the most extreme point within 1.5 times the IQR. Outliers are not shown for clarity.

© The Author(s)

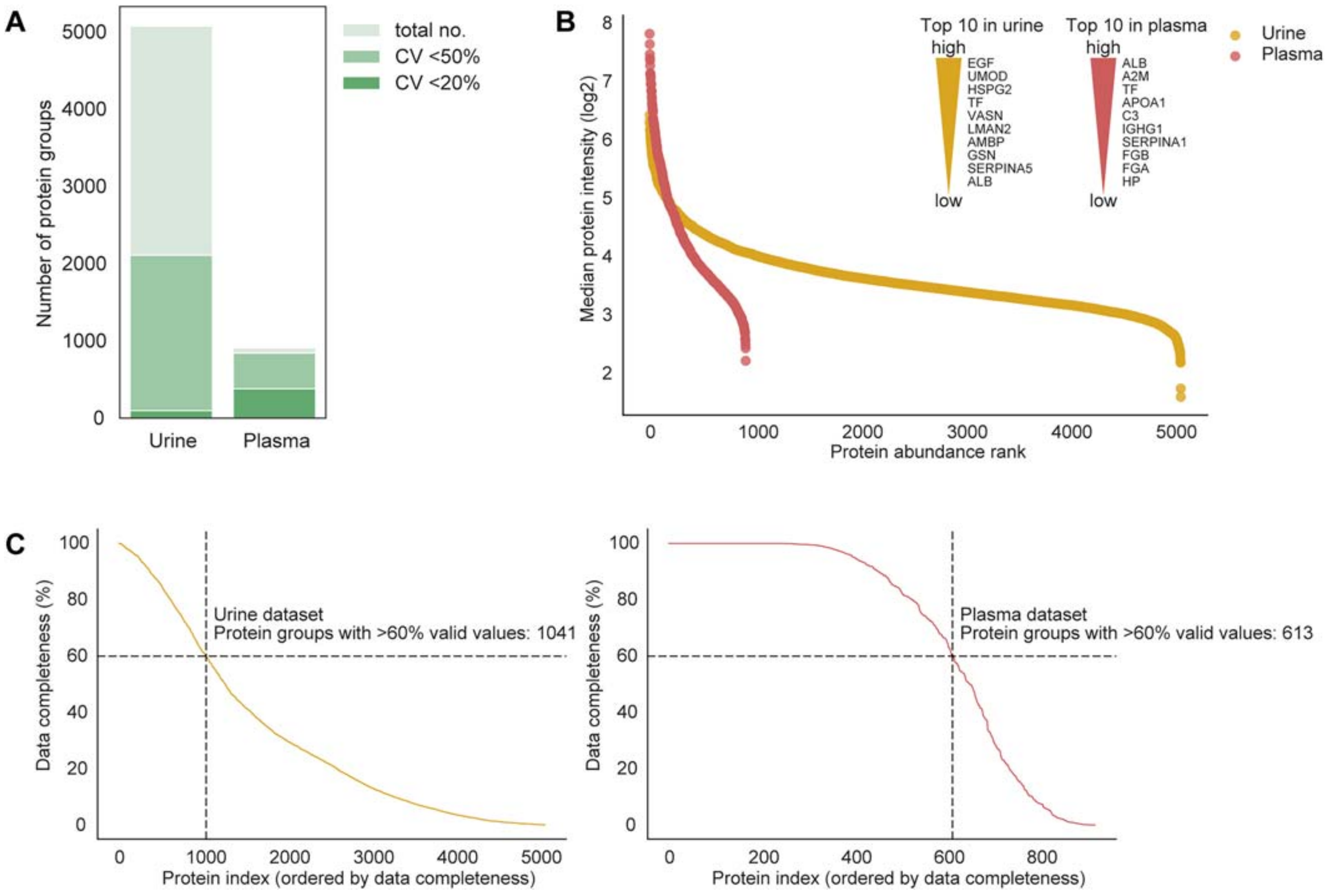

Figure EV2. Proteomic profiling of urine and neat plasma samples in a pediatric cohort.

(**A**) Cumulative protein group identifications in each body fluid. Proportion of protein groups with <50% and <20% biological CV for both is shown. (**B**) Abundance rank plot of protein groups based on median protein intensities. The top ten most abundant protein groups for each body fluid are listed. (**C**) Data completeness curve for each body fluid (left: urine; right: neat plasma). Number of protein groups quantified with >60% data completeness is shown.

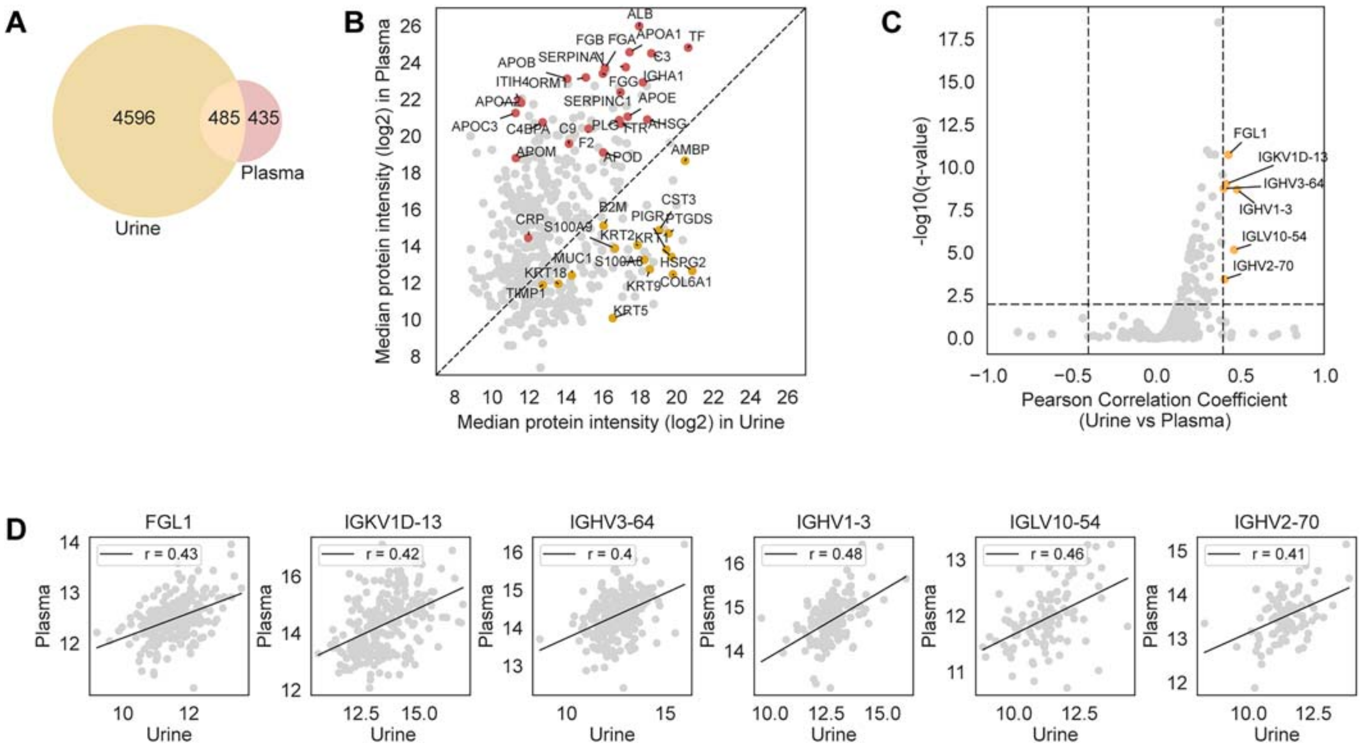

Figure EV3. Integration of urine and neat plasma proteome data.

(**A**) Venn diagram showing distinct protein group identifications in the urine and neat plasma proteomes, or in both. (**B**) Abundance map of the commonly identified proteins in the urine and neat plasma proteomes, showing the correlation between their median protein intensities in log2 space. Apolipoproteins, complement system proteins, coagulation factors, and other known plasma proteins are highlighted in red. Proteins related to kidney function and filtration, as well as structural and epithelial proteins such as keratins and mucins are highlighted in yellow. A diagonal line representing $x = y$ is shown as a gray, dashed line. (**C**) Volcano plot displaying Pearson correlation coefficients between protein intensities in the urine and neat plasma proteome across the cohort, along with their associated FDR-adjusted P values (Benjamini–Hochberg correction). Statistically significant, moderately correlating proteins (FDR-corrected P value < 0.01 and Pearson's $r \geq 0.4$) are highlighted. (**D**) Correlation plots of log2-transformed protein intensities of the urine and neat plasma proteomes for each individual protein highlighted in (**C**). Regression lines are shown as solid black lines, and the Pearson's r values are displayed.

 © The Author(s)

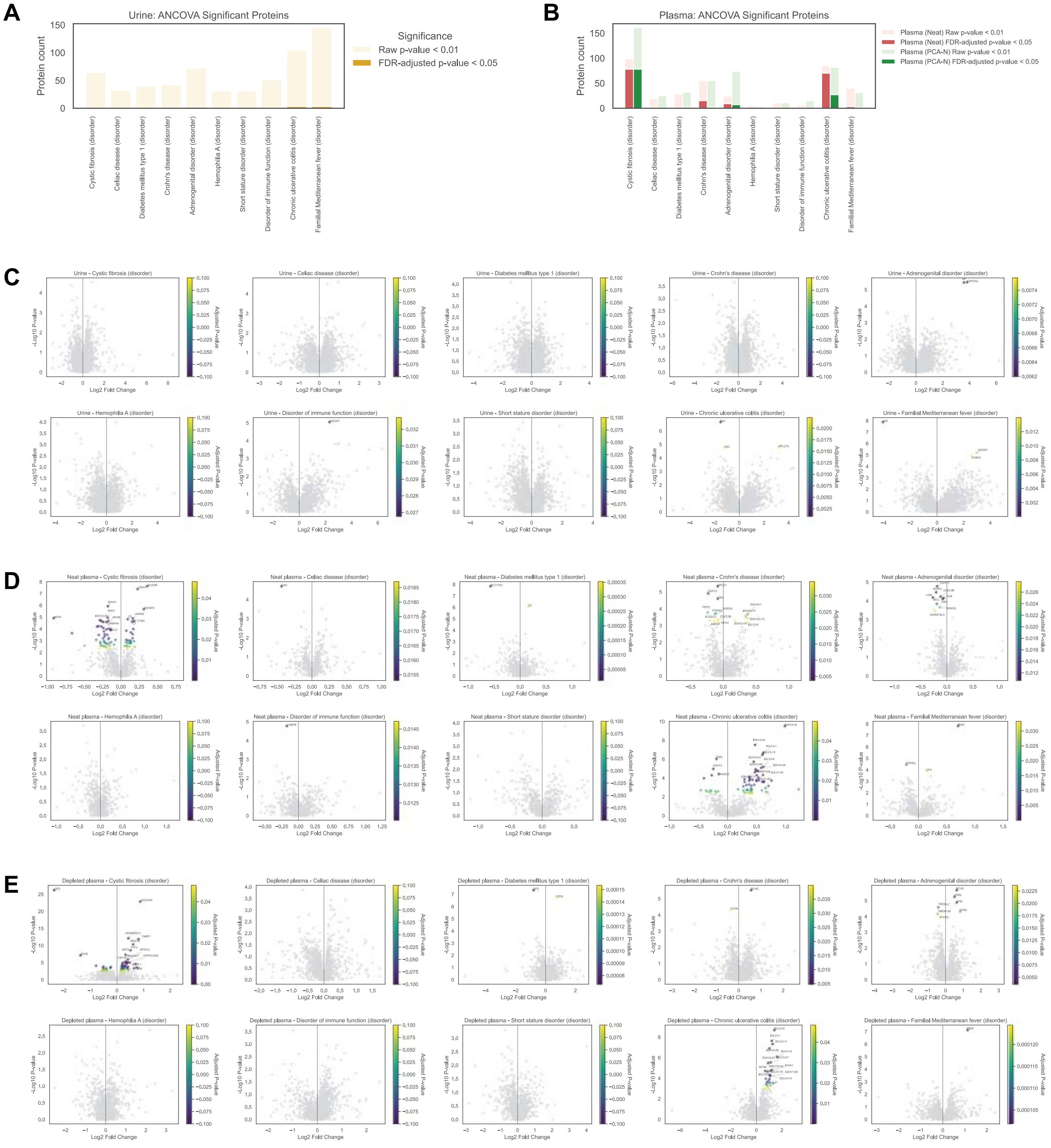
A
Urine: ANCOVA Significant Proteins
Protein count
Significance
Raw p-value < 0.01
FDR-adjusted p-value < 0.05
Cystic fibrosis (disorder)
Celiac disease (disorder)
Diabetes mellitus type 1 (disorder)
Crohn's disease (disorder)
Adrenogenital disorder (disorder)
Hemophilia A (disorder)
Short stature disorder (disorder)
Disorder of immune function (disorder)
Chronic ulcerative colitis (disorder)
Familial Mediterranean fever (disorder)
B
Plasma: ANCOVA Significant Proteins
Plasma (Neat) Raw p-value < 0.01
Plasma (Neat) FDR-adjusted p-value < 0.05
Plasma (PCA-N) Raw p-value < 0.01
Plasma (PCA-N) FDR-adjusted p-value < 0.05
C
D
E
Log2 Fold Change
-Log10 P-value
Adjusted P-value

◀ **Figure EV4. Differential expression analysis of the top ten most prevalent diseases versus healthy controls.**

(**A**) Number of differentially regulated protein groups (raw *P* value < 0.01) in the urine proteome for each disease in the top ten most prevalent diseases, as identified by ANCOVA with age and sex as covariates. Highlighted in darker yellow color is the number of differentially regulated protein groups that have an FDR-adjusted (Benjamini–Hochberg correction) *P* value < 0.05. (**B**) Number of differentially regulated protein groups (*P* value < 0.01) in the plasma proteome for each disease in the top ten most prevalent diseases, as identified by ANCOVA with age and sex as covariates. The neat plasma dataset measured on the Bruker timsTOF HT is shown in red, while the PCA-N plasma dataset measured on the Thermo Orbitrap Astral is shown in green. Highlighted in darker colors are the number of differentially regulated protein groups that have an FDR-adjusted (Benjamini–Hochberg correction) *P* value < 0.05. (**C**) Representative volcano plots illustrating differential protein expression for each disease compared to healthy controls in the urine proteome, analyzed using ANCOVA with sex and age as covariates. Note that the volcano plot for adrenogenital disorder is also shown in Fig. 3E. (**D**) Representative volcano plots illustrating differential protein expression for each disease compared to healthy controls in the neat plasma proteome, analyzed using ANCOVA with sex and age as covariates. Note that the volcano plot for cystic fibrosis is also shown in Fig. 3A. (**E**) Representative volcano plots illustrating differential protein expression for each disease compared to healthy controls in the PCA-N plasma proteome, analyzed using ANCOVA with sex and age as covariates. Note that the volcano plot for cystic fibrosis is also shown in Fig. 3C.

 © The Author(s)

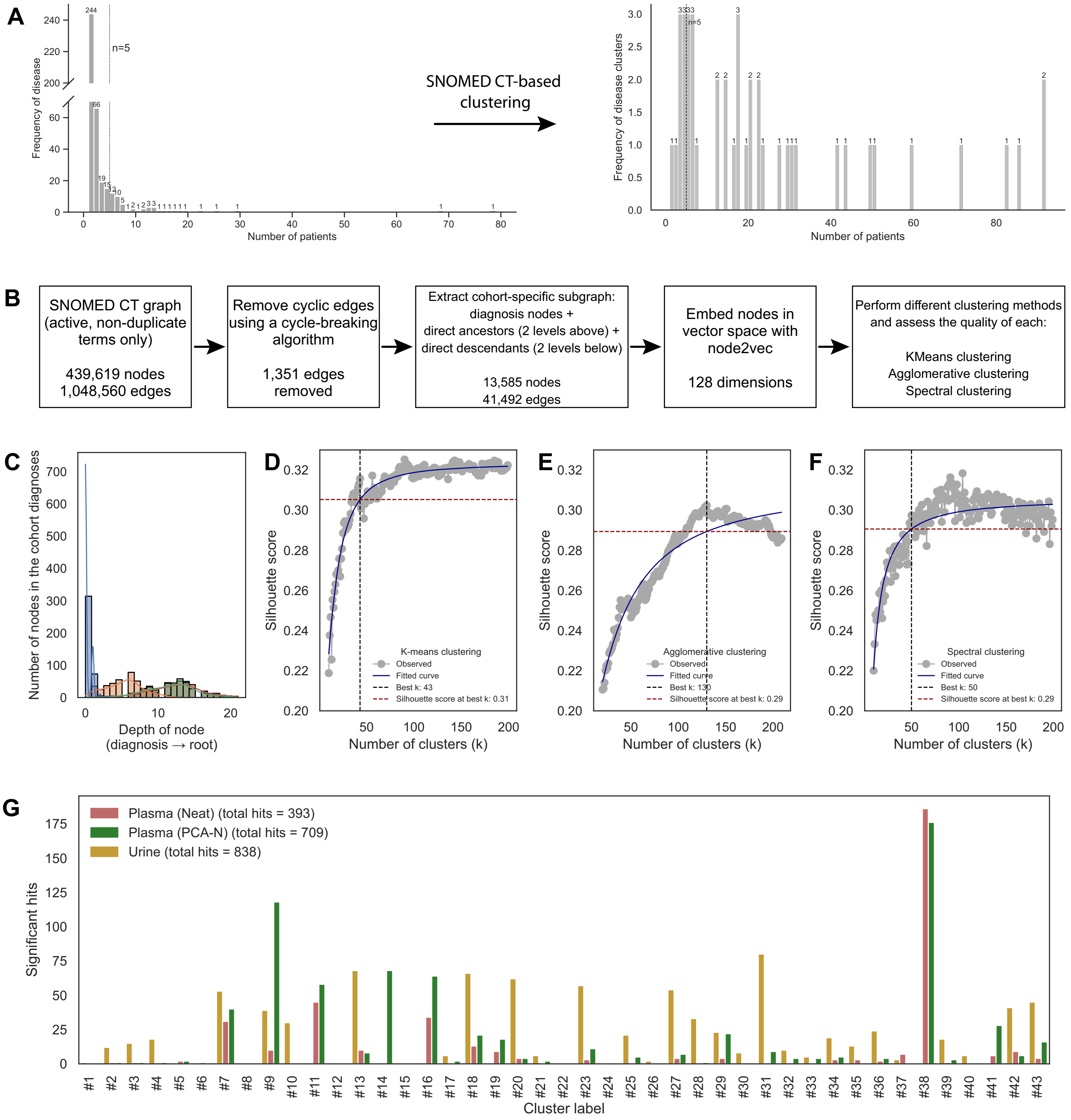
A
Frequency of disease
n=5
Number of patients
SNOMED CT-based clustering
Frequency of disease clusters
n=5
Number of patients
B
SNOMED CT graph (active, non-duplicate terms only)
439,619 nodes
1,048,560 edges
Remove cyclic edges using a cycle-breaking algorithm
1,351 edges removed
Extract cohort-specific subgraph: diagnosis nodes + direct ancestors (2 levels above) + direct descendants (2 levels below)
13,585 nodes
41,492 edges
Embed nodes in vector space with node2vec
128 dimensions
Perform different clustering methods and assess the quality of each:
KMeans clustering
Agglomerative clustering
Spectral clustering
C
Number of nodes in the cohort diagnoses
Depth of node (diagnosis → root)
D
Silhouette score
K-means clustering
Observed
Fitted curve
Best k: 43
Silhouette score at best k: 0.31
Number of clusters (k)
E
Silhouette score
Agglomerative clustering
Observed
Fitted curve
Best k: 130
Silhouette score at best k: 0.29
Number of clusters (k)
F
Silhouette score
Spectral clustering
Observed
Fitted curve
Best k: 50
Silhouette score at best k: 0.29
Number of clusters (k)
G
Plasma (Neat) (total hits = 393)
Plasma (PCA-N) (total hits = 709)
Urine (total hits = 838)
Significant hits
Cluster label

© The Author(s)

Figure EV5. Construction and clustering of SNOMED CT ontology network for disease grouping.

(**A**) Distribution of patient counts across disease categories before and after SNOMED CT-based clustering. The main histogram shows the number of original disease categories in the cohort (prior to clustering), binned by patient count. The dashed vertical line at $n = 5$ indicates the threshold for identifying the number of disease groups containing less than five patients. The inset displays the corresponding distribution after clustering, demonstrating a marked reduction in the number of small patient groups. (**B**) Schematic of the SNOMED CT-based clustering pipeline. (**C**) Depth from diagnosis node to root node across different inclusion windows (1, 2, or 3 levels of ancestors and descendants). For each subgraph, we calculated the maximum upward path length from each cohort diagnosis node to the most distant ancestor. (**D**) Silhouette score curves across values of k (number of clusters) ranging from 10 to 200 for k-means clustering. The best k was defined as the point where the Silhouette score reached 97% of its maximum. (**E**) Same as in (**D**) but for agglomerative clustering. (**F**) Same as in (**D**) but for spectral clustering. (**G**) Total number of significantly altered protein groups identified per body fluid after ontology-guided disease clustering. Bar plots show the number of differentially expressed proteins in each proteomics dataset—urine, neat plasma, and PCA-N plasma—compared to healthy controls. Significance was determined using Welch's *t* test with Benjamini–Hochberg correction (FDR < 0.05).

 © The Author(s)
